# Complete Data Analysis Workflow for Quantitative DIA Mass Spectrometry Using Nextflow

**DOI:** 10.1021/acs.jproteome.5c00266

**Published:** 2026-02-06

**Authors:** Mats Perk, Sami Pietilä, Tommi Välikangas, Balazs Balint, Tomi Suomi, Laura L. Elo

**Affiliations:** † Turku Bioscience Centre, University of Turku and Åbo Akademi University, FI-20520 Turku, Finland; ‡ Institute of Biomedicine, 8058 University of Turku, FI-20520 Turku, Finland

**Keywords:** mass spectrometry, data-independent acquisition, nextflow, data analysis, quantitative proteomics

## Abstract

Data-independent acquisition (DIA) mass spectrometry is a technique used in proteomics to identify and quantify proteins in complex biological samples. While this comprehensive approach yields more complete and reproducible protein profiles than data-independent acquisition (DDA), the resulting data are substantially larger and more complex, presenting significant challenges for data analysis and interpretation. These challenges can be effectively addressed using dedicated workflow managers that support parallel execution of complex analysis pipelines on high-performance computing infrastructure. Nextflow, in particular, is well-suited for streamlining data analysis, as it automates key aspects of workflow management, allowing researchers to efficiently analyze large-scale data sets with minimal manual intervention. Here, we describe glaDIAtor-nf, a Nextflow version of our software package glaDIAtor for untargeted analysis of DIA mass spectrometry proteomics data. We first demonstrate its technical accuracy through rigorous testing on gold standard data sets. Building on this, we then reveal known proteome patterns from public breast cancer data that remained hidden in the processed data of the original study. This illustrates the potential of reanalyzing the accumulating public data, but also highlights the need for convenient tools to facilitate such reanalysis in large-scale.

## Graphical abstract

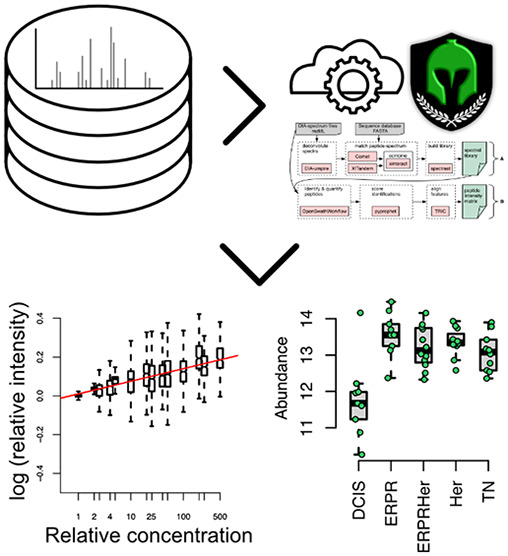

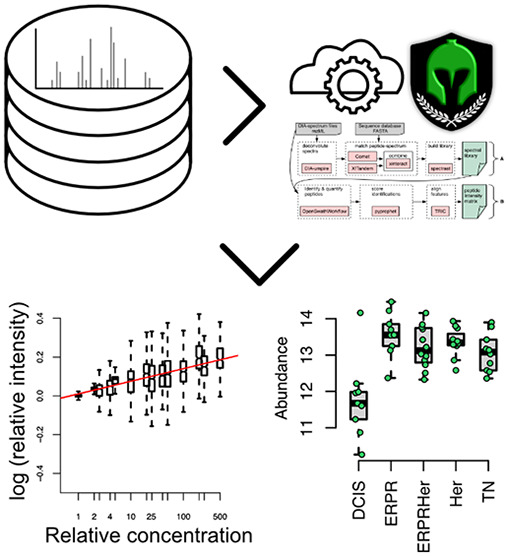

## Introduction

Proteomics is a field of study that aims to understand the properties and functions of proteins within biological systems.^1^ A key aspect of mass spectrometry proteomics is data analysis, which involves identification and quantification of proteins, as well as post-translational modifications.^2^ Modern proteomics has the capacity to considerably improve our understanding of biological systems and, for instance, underlying disease mechanisms, aiding the development of new diagnostic and therapeutic strategies for complex diseases.^3,4^

One specific high-throughput proteomic method is data-independent acquisition (DIA) mass spectrometry, where the mass spectrometer systematically scans over a wide range of *m*/*z* values,^5^ resulting in more accurate and reproducible quantification of protein intensities compared to data-dependent acquisition (DDA) method.^6^ DIA analysis is particularly useful in complex biological systems, where the number of proteins can be vast, and for large-scale studies, where the number of samples is large.^7,8^ This is because the DIA mass spectrometry allows for the analysis of all peptides present in a sample regardless of their abundance, providing a more comprehensive and accurate characterization of the proteome than the widely used DDA approach, which has a semistochastic ion selection. However, the analysis of DIA data is more challenging due to its high complexity, requiring advanced computational tools and algorithms to extract meaningful insights.

Over the years, a wide range of software tools have been developed for the analysis of DIA data. Notable examples include the commercial Spectronaut,^9^ the commercial Peaks software (including deepnovo-dia^10^), the dual-licensed (i.e., *gratis* for academia, commercial for all other use) DIA-NN^11^ and FragPipe,^13^ and the *gratis* MaxDIA^12^ (part of the MaxQuant toolkit). It is worth noting that all the tools above are closed source, limiting the ability of researchers to review or adapt the underlying algorithms. To promote open and reproducible research, there is a clear need for free and open source (as in *libre*) analysis software.

Many DIA analysis tools are characterized by a strong emphasis on user-friendly, interactive use. They are typically installed on a single workstation and operated through intuitive graphical user interfaces. While this design is convenient for most users, it presents significant challenges when attempting to scale these tools beyond a single workstation or integrate them into custom analysis pipelines.

To address these limitations, several workflow management systems have been developed with scalability and massively parallel execution in mind. Examples include Galaxy,^14^ Snakemake,^15^ Common Workflow Language (CWL),^16^ and Nextflow.^17^ These systems streamline data processing by automatically allocating computational resources, managing error handling and recovery, and ultimately enabling robust and reproducible data analysis. The nf-core project has curated a collection of Nextflow-based analysis pipelines, making it a valuable resource for researchers.^18^ Some tools that were originally released as standalone software, such as FragPipe and MaxQuant, have since been adapted to run under Galaxy or Nextflow (e.g., nf-encyclopedia^19^). Others, including quantms,^20^ were developed specifically within the Nextflow workflow management system.

The objective of this work was to develop a free and open source, robust, versatile data analysis workflow for untargeted analysis of DIA mass spectrometry data, spanning from the analysis of single-organism proteomes to complex metaproteome analyses, using Nextflow as the underlying workflow management infrastructure. Nextflow is particularly well-suited for proteomic data analysis workflows, as it allows for the handling of complex and dynamic workflows in a reproducible and scalable manner. Additionally, Nextflow enables easy integration with various bioinformatics tools and resources, making data analysis more accessible to researchers with varying levels of bioinformatics expertise. Furthermore, Nextflow is an ideal platform for research infrastructures such as ELIXIR, where it has been widely deployed.^21^

To this end, we introduce here glaDIAtor-nf, a free and open-source Nextflow version of our software package glaDIAtor,^22^ with the aim to assist researchers in their DIA mass spectrometry proteomics studies. We demonstrate the performance of our workflow using publicly available gold standard data sets, providing a benchmark for its practical application. Finally, we demonstrate its ability to reveal new information from publicly available resources beyond the original studies, with an example from a breast cancer proteome study.

## Materials and Methods

### Data Sets

We analyzed three publicly available gold standard technical data sets in this study; two spike-in and one mixture data set. The first spike-in data set is from Bruderer et al.^9^ (PASS00589), where three different mixes from 12 nonhuman proteins are added to a constant human background (HEK-293), forming eight different spike-in concentrations, each with three technical replicates. The second spike-in data set is from Gotti et al.^23^ (PXD026600), where Universal Proteomics Standard (UPS1), consisting of 48 human proteins, is added to *Escherichia coli* background, forming eight different spike-in concentrations, each with three technical replicates. The file RD139_Narrow_UPS1_50fmol_inj3.raw was excluded as it was found to be corrupted. The third technical data set is a multispecies sample mixture by Jumel at al.^24^ (MSV000090837), containing two groups of samples with different ratios of *E. coli*, human and yeast, each with four technical replicates.

We also analyzed real-world data from Valo et al.^25^ (PXD014194) consisting of tissue samples from 52 individuals with different subtypes of breast cancer and 20 individuals with noninvasive ductal carcinoma in situ (DCIS).

The Bruderer, Gotti, and Valo data sets were first converted from proprietary formats to the open mzML format using proteowizard 3.0.22167-d016184. The DDA data from the Valo data set was converted to mzXML format with proteowizard. For the Jumel data set, the mzML files were obtained directly from its MassIVE repository.

### Performing the glaDIAtor-nf Analysis

We installed the glaDIAtor-nf workflow (tagged ‘analysis’) to a high performance computing cluster consisting of six computing nodes, each with over 200 GB of RAM memory and over 64 CPU cores. For each data set, we executed the workflow to produce peptide and protein intensity tables as tsv-files.

Peptide search for the Bruderer data was performed against the UniProtKB database of Human (2017/04, 20183 protein entries), appended with sequences of nonhuman spike-in proteins. Peptide search for the Gotti data was performed against the database provided in its respective MassIVE repository, which is a UniProtKB database of *E. coli* (2016/03, 4314 protein entries), appended with 48 UPS1 spike-in proteins. Peptide search for the Jumel data was performed against the UniProtKB database of *E. coli*, human, and yeast, using the same databases as the original publication (2020/08, 30810 total proteins). The Valo breast cancer data was searched against UniProtKB Human database 2016/02 (20199 protein entries) matching the original publication, and a more recent database dated 2024/10 (20429 protein entries) to assess the effect of the database version.

Peptide identifications were controlled by applying a false discovery rate (FDR) of 1%. The Gotti data set was scored with legacy pyprophet version 0.24.1 (--pyprophet_use_legacy = true parameter), while the other data sets were analyzed with pyprophet 2.2.5. The Valo data set was analyzed with fragment mass tolerance of 0.350 Da and a precursor mass tolerance of 50 ppm in DDA-assisted mode. All other data sets were analyzed with a fragment mass tolerance of 0.02 Da and a precursor mass tolerance of 10 ppm. Searches were performed with trypsin as the digestion enzyme, with one allowed miscleavage. Minimum length for peptides was set to 5 and maximum to 63. The carbamidomethylation of cysteine was set as a fixed modification and the oxidation of methionine as a variable modification. Transfer of identification confidence (TRIC) alignment was set to 1% target FDR and 5% maximum FDR for all data sets; the Valo data set using the diRT realignment method, and the Gotti and Jumel using the linear realignment method.

Additional information about usage of the glaDIAtor-nf workflow, including the process for conducting a DDA-assisted analysis, is outlined in the glaDIAtor-nf user manual, which is available on the GitHub repository (https://github.com/elolab/glaDIAtor-nf).

### Statistical Analysis

Protein abundances were normalized using variance stabilizing normalization (VSN)^26^ and protein abundances of zero were replaced with missing values (*NA*).

With the Gotti, Jumel, and Valo data sets, we confirmed that the changes in protein intensities produced by glaDIAtor-nf corresponded to the known changes in the concentrations between the samples. For every pairwise combination of injections, we calculated the logarithmic fold change of observed protein abundances and the known concentrations for each spike-in or mixture proteins. We subsequently calculated the Pearson correlation between these variables. For differential expression analysis, ROTS (version 1.3.0)^27^ was applied for all sets of two or more sample groups (i.e., 2^*n*^–1–*n* comparisons, where *n* is the number of biological samples), with 1000 bootstrap resamples (*B*), and a maximum top list size (*K)* of 5000. A protein was included in the ROTS analysis if it was observed in at least two replicates in each sample group of that comparison. The ROC curves were determined over the concatenation of the resulting ROTS *p*-values as predictor and the spike-in status of the protein as response. True positives are proteins that are known to differ in concentration between conditions and false positives are proteins that are not expected to differ but are still identified as differentially expressed.

### Nextflow

glaDIAtor-nf software was implemented with Nextflow version 21.04.3, which supports the Domain Specific Language 1 (DSL1) syntax. We selected this engine to leverage its distinct advantages over other workflow management engines such as Snakemake. It is inherently designed for high-performance computing environments, offering broad compatibility with a variety of job schedulers and compute platforms, which ensures exceptional code portability. To maximize robustness and reproducibility, Nextflow also provides native integration with various containerization technologies such as Docker, Apptainer, and Podman. Furthermore, it features automatic execution tracing and supports seamless resumption of interrupted runs, enhancing workflow reliability and efficiency.

### Containers

The dependencies of glaDIAtor-nf are distributed as Open Container Initiative^28^ and Apptainer^29^ containers, created with GNU Guix.^30^ Nextflow will automatically retrieve these containers from the public repository when the workflow is run.

### Compute Performance

In Table 1, “CPU time” is the sum of the duration of all Nextflow processes. To account for caching in reruns, wall time was calculated by taking the start and end time per process, merging overlapping time ranges, and then summing the durations over all time ranges. Sequential average CPU usage was calculated by weighing the CPU usage by the process time.

**Table 1:** Summary of Resource Usage^a^

Data set Name	Wall-clock time	CPU time	Peak RAM (GiB)	CPU Usage (%)	Number of sequences in FASTA	Number of DDA/Pseudospectra	Number of DIA Spectra
Gotti	5 h 40 min	57 h 56 min	54.08	1636.2	4362	3,269,888	3,342,328
Bruderer	10 h 10 min	207 h 28 min	54.37	3421.5	20,199	9,697,064	1,061,755
Jumel	5 h 9 min	48 h 27 min	54.27	1480.4	31,055	1,790,536	1,235,515
Valo^b^	5 h 4 min	117 h 41 min	46.09	749.9	20,432	829,001	19,834,290

a Wall-clock time represents the total elapsed time from the start to the completion of the job. CPU time denotes the cumulative time spent on computation by all CPU cores. The size of a FASTA file is determined by the number of sequences it contains.

b Used DDA-data to build a spectral library.

### DIA-NN Analysis

DIA-NN (version 1.8.1) was used to reanalyze three gold-standard public data sets (Bruderer et al.^9^ PASS00589, Gotti et al.^23^ PXD026600, and Jumel et al.^24^ MSV000090837). Reference sequence versions and DIA-NN execution parameters are detailed in the Supplementary Materials and Methods.

## Results

### Quantitative glaDIAtor-nf DIA Data Analysis Workflow

The quantitative DIA data analysis workflow of glaDIAtor-nf is outlined in Figure 1. The input for the workflow includes DIA spectrum files and a protein sequence database to be used as the search space for all possible proteins present in the samples. The workflow can be divided into two primary phases: (A) the construction of a reference spectral library, and (B) the identification and quantification of peptides using the generated reference spectral library. For untargeted analysis of DIA data, phase A involves the construction of a pseudospectral library. This includes deconvolution of the spectra, followed by peptide-spectrum matching, and generation of the library based on the identified peptide spectra. Then, in the subsequent phase B, peptides are identified and quantified from the DIA spectrum files using the pseudospectral library, incorporating identification scoring and feature alignment steps to produce a peptide intensity matrix.

**Figure 1:**
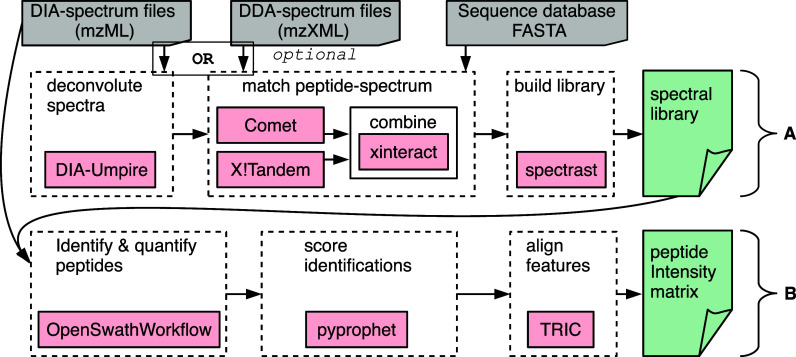
Schematic diagram of glaDIAtor-nf workflow. The workflow is divided into two main parts. In the A section a spectral library is generated, either from DIA data deconvoluted by DIA-Umpire, or from external DDA spectra. Then, in the B section peptides are identified based on the library generated in the previous section, followed by quantification.

In addition to the untargeted analysis using pseudospectral libraries, the glaDIAtor-nf workflow also supports DDA-assisted DIA analysis. In that case, the workflow utilizes DDA data files to construct a reference spectral library, instead of constructing a pseudospectral library. The use of a spectral library constructed from high-quality DDA data acquired through multiple injections of the same sample can improve the efficiency of peptide identification, resulting in the detection of a larger number of peptides.^22^

The implementation of the workflow utilizing Nextflow is rooted in the principles of automated execution of the data analysis steps and provision of a suitable execution environment with the necessary programs and utilities in each step. This approach facilitates a user-friendly experience, as it only requires the setup of data and adjustment of the instrument- and experiment-specific parameters, such as precursor and fragment tolerances and false discovery rate thresholds for peptide identification, prior to initiating the analysis. Furthermore, glaDIAtor-nf is compatible with various container technologies and executors, including SLURM, thereby enabling the execution of the workflow in a variety of high-performance computing environments.

As an illustration, the following command can be utilized to launch the glaDIAtor-nf analysis using Nextflow:
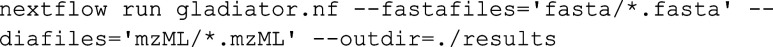


Upon completion of the analysis, the results are located in the designated output directory (--outdir).

### Assessment with Gold Standard Benchmark Data Sets Confirms High Accuracy

To assess the technical accuracy of glaDIAtor-nf, we used three gold standard data sets: two spike-in data sets and a multispecies mixture data. The Bruderer spike-in data set^9^ contained eight samples with three technical replicates each and 12 spike-in proteins divided over three mixes in different concentrations to a human background. The Gotti spike-in data set^23^ included eight samples with three technical replicates each and 48 proteins spiked into a background proteome of *E. coli*, in increasing concentrations. The Jumel multispecies mixture data set^24^ contained two sample groups with four replicates each, composed of yeast, human, and *E. coli* with known differences between the sample groups.

In the Bruderer spike-in data set, glaDIAtor-nf identified a total of 11336 peptides and 2531 protein groups, of which 2049 had only a single member protein, including all 12 spike-in proteins. The peptide and protein intensity data had 0.4 and 0.1% of missing values, respectively. In the Gotti spike-in data set, glaDIAtor-nf identified a total of 8122 peptides, 1393 protein groups, and 1361 single proteins, including 46 out of the 48 spike-in proteins. The peptide and protein intensity data had 0.1 and 0.03% of missing values, respectively. In the Jumel multispecies mixture data set, glaDIAtor-nf identified a total of 18814 peptides, 4749 protein groups, and 3903 single proteins, including 1122 yeast, 303 *E. coli*, and 2478 human proteins. The peptide intensity data matrix had 0.04% of missing values and there were no missing values at the protein level.

To investigate how the intensities reported by glaDIAtor-nf corresponded to the known changes in the spike-in and mixture concentrations, we calculated Pearson correlation between the logarithmic fold-changes of the spike-in protein intensities and the logarithmic fold changes of their known concentrations between the sample groups. Importantly, we observed highly significant correlations: 0.93 for the Bruderer data set (*p* < 0.01), 0.56 for the Gotti data set (*p* < 0.01), and 0.84 for the Jumel data set (*p* < 0.01) (Figure 2A–C). These results confirmed that increased relative concentration of proteins corresponded to increased protein intensities quantified by glaDIAtor-nf.

**Figure 2:**
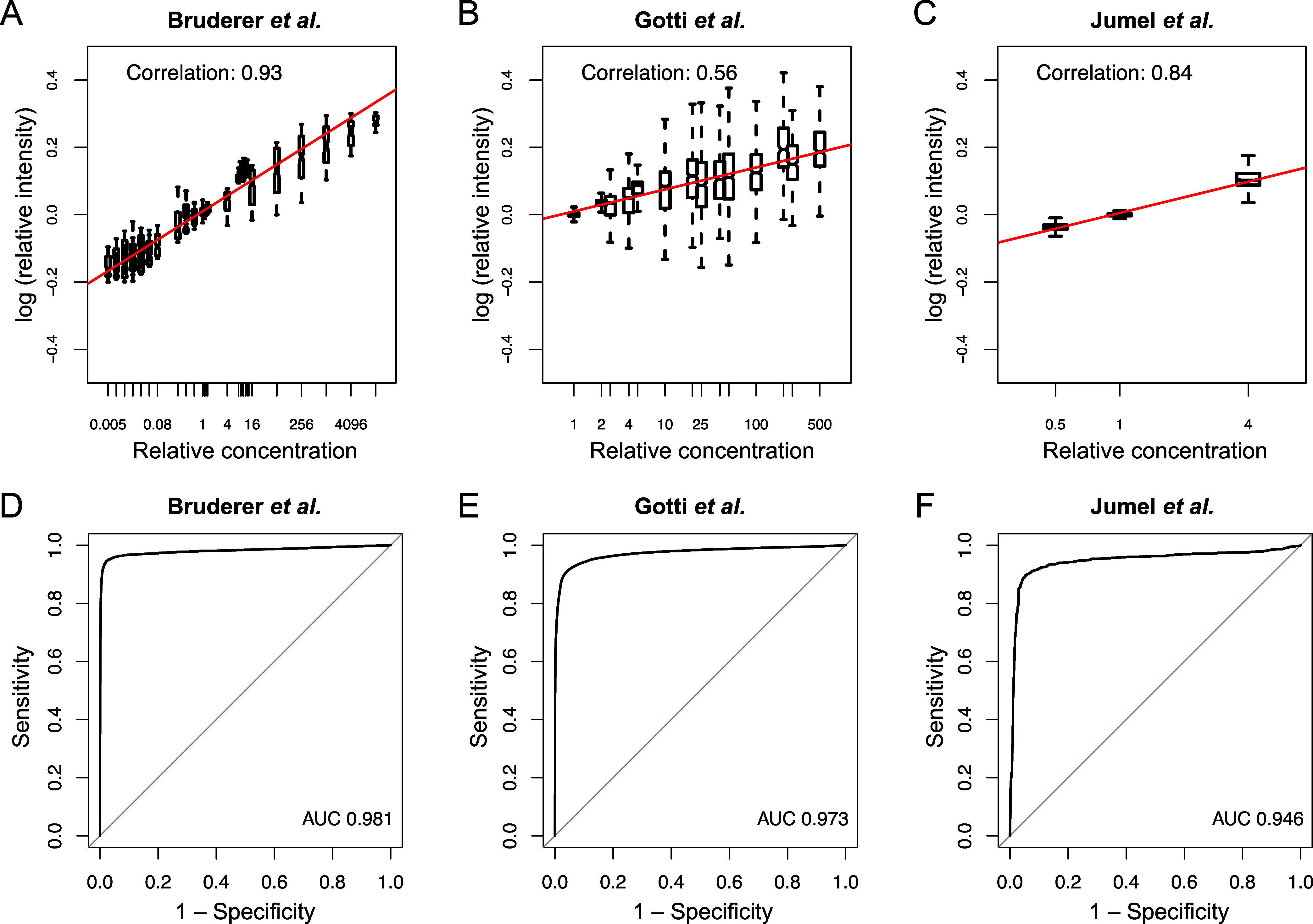
Benchmark of glaDIAtor-nf in gold standard data sets. Correlations between known relative concentrations of the spike-in proteins and their intensities quantified by glaDIAtor-nf in (**A**) Bruderer, (**B**) Gotti, and (**C**) Jumel data sets. The receiver operating characteristic (ROC) curves over all possible pairwise comparisons in (**D**) Bruderer, (**E**) Gotti, and (**F**) Jumel data sets together with the areas under the ROC curves (AUCs).

To further demonstrate the utility of glaDIAtor-nf for quantitative analysis of differential expression, we performed pairwise and multigroup analysis using the reproducibility-optimized ROTS approach.^27^ Assessment of the areas under the receiver operating characteristic (ROC) curves (AUC) revealed high sensitivity and specificity of the findings, with AUC of 0.98 for the Bruderer, 0.97 for the Gotti, and 0.95 for the Jumel data set, respectively (Figure 2D–F). For comparison, we also applied the same analysis to data processed with DIA-NN, a widely used standalone DIA analysis tool that is based on the use of in silico spectra generated from a reference protein database through machine learning. The results obtained with DIA-NN were comparable to those obtained with glaDIAtor-nf (Figure S1). Altogether, our results indicate that glaDIAtor-nf produces reliable results for differential expression analysis.

### Reanalysis of Existing Data Can Lead to Novel Findings

To demonstrate the utility of glaDIAtor-nf in analyzing and reanalyzing real data sets, we used SWATH-MS DIA data by Valo et al.,^25^ available from the PRoteomics IDEntifications database PRIDE, including tissue samples from 52 individuals with breast cancer, and an additional 20 individuals with noninvasive ductal carcinoma in situ (DCIS). The breast cancer samples were further divided into estrogen/progesterone receptor positive cases (ERPR), estrogen/progesterone/HER2 positive cases (ERPRHer), HER2 positive cases (Her), and triple negative cases (TN). In the reanalysis, glaDIAtor-nf identified 862 protein groups, of which 703 consisted of a single protein.

Sample-wise Pearson correlations over protein abundances between the original and glaDIAtor-nf processed data were highly significant (*p* < 0.001), ranging from 0.76 (Figure 3A) to 0.47 depending on the sample. Considering only uniquely identified proteins, a multigroup differential expression analysis between the different breast cancer subtypes (ERPR, ERPRHer, Her, TN) and DCIS using reproducibility-optimized test-statistic ROTS identified 194 proteins with the glaDIAtor-nf processed data, compared to 104 proteins detected using the data processed in the original study (false discovery rate FDR < 0.05, Figure 3B). The reanalyzed data was searched against the UniProt (Swiss-Prot) database dated 2016/02 to match the database used in the original analysis. Principal component and correlation analysis (Figure S2) supported that the protein quantification matrix processed with glaDIAtor-nf enabled a clear separation particularly between noninvasive DCIS and invasive breast cancer subtypes.

**Figure 3:**
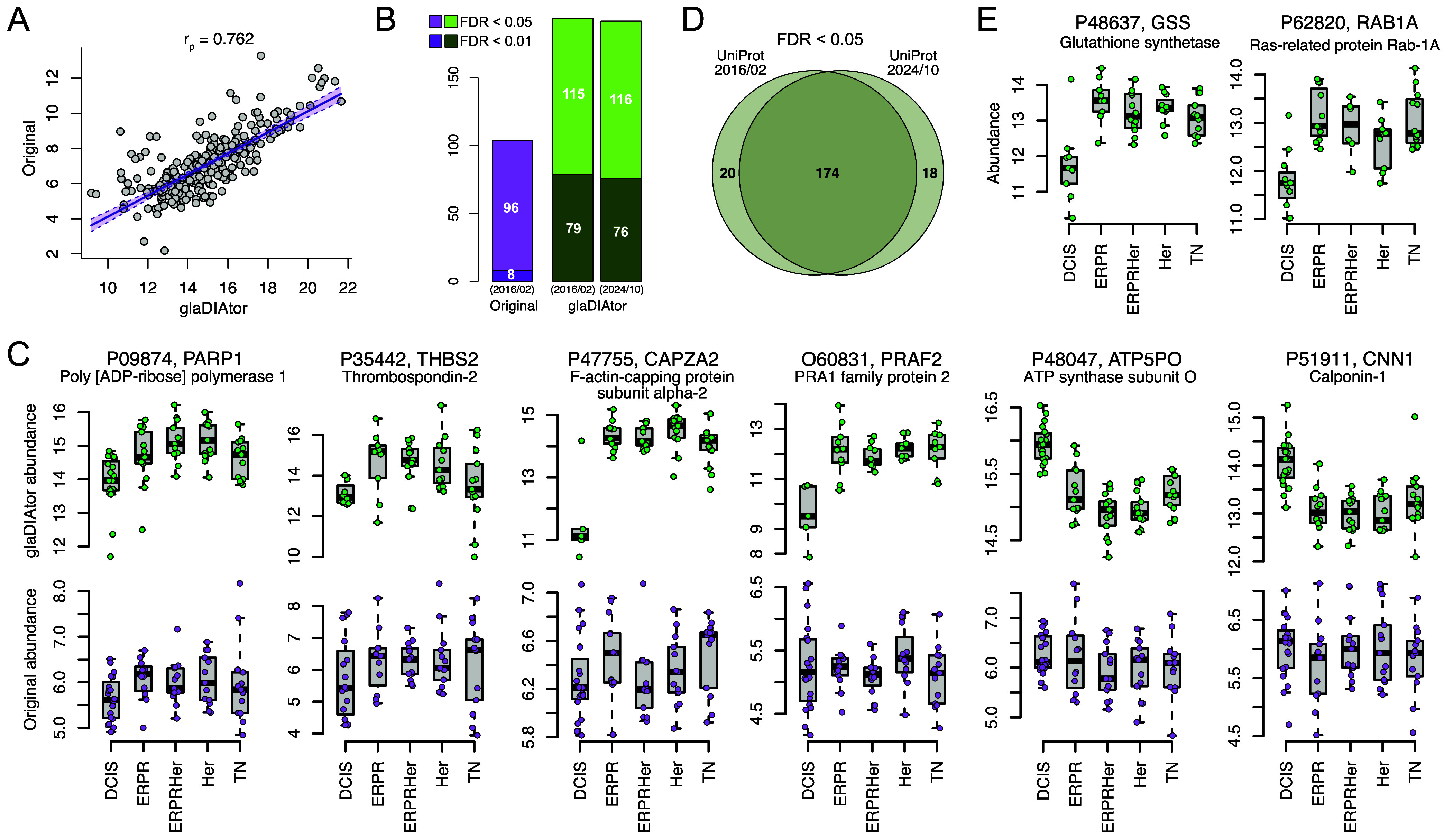
Application of glaDIAtor-nf in clinical proteomics data on breast cancer. (**A**) Correlation of protein abundances between the original and glaDIAtor-nf processed data. Sample with the highest Pearson correlation (*r* = 0.762) is shown. (**B**) Number of differentially expressed proteins (FDR < 0.05) between different breast cancer subtypes (ERPR, ERPRHer, Her, TN) and noninvasive ductal carcinoma in situ (DCIS) in the original and reanalyzed data. (**C**) Differentially expressed proteins between breast cancer subtypes and DCIS, where the difference is detected only using glaDIAtor-nf (FDR < 0.05 in the reanalyzed data and FDR > 0.5 in original data), using the UniProt database from 2016/02 that matches the original analysis. (**D**) Overlap of differentially expressed proteins between breast cancer subtypes and DCIS (FDR < 0.05) when using the UniProt database from 2016/02 that matches the original analysis or a recent version from 2024/10. (**E**) Differentially expressed proteins between breast cancer subtypes and DCIS found only when using the recent UniProt database from 2024/10 and not with the UniProt database from 2016/02.

Investigation of the differentially expressed proteins revealed six proteins that were highly significant in the glaDIAtor-nf reanalysis (FDR < 0.01), but clearly not significant when using the original processed data (FDR > 0.5). These included poly­[ADP-ribose] polymerase 1 (PARP1), thrombospondin-2 (THBS2), F-actin-capping protein subunit α-2 (CAPZA2), and PRA1 family protein 2 (PRAF2), whose abundance was higher in the breast cancer subtypes compared to DCIS, and ATP synthase subunit O (ATP5PO), and calponin-1 (CNN1), whose expression was highest in DCIS (Figure 3C). *PARP1* expression has been found to be significantly increased in several primary malignancies, including breast cancer,^31^ consistent with the findings here when compared to DCIS. *THBS2* has been suggested to promote cancer progression^32^ and is considered as a functional biomarker for patients with triple-negative breast cancer.^33^ *CAPZA2* has been found as differentially mutated between ER+ and ER- breast cancer subtypes,^34^ whereas *PRAF2* has been found to promote the proliferation and invasion of breast cancer cells.^35^ The Human Protein Atlas^36^ reports reduced ATP5PO levels for several cancers compared to healthy tissues. On the other hand, high gene expression levels of the mitochondrial ATP synthase composing proteins, such as *ATP5PO*, have been found in cancer and linked to poor prognosis,^37^ but also the overexpression of *ATP5PO* has specifically been linked to reduced ATP5PO protein levels^38^ in zebrafish. Similarly, *CNN1* has been observed to have higher expression in normal tissues compared to tumor tissues across most cancer types,^39^ in line with our results here.

In addition to using the UniProt (Swiss-Prot) database dated 2016/02 that matched the database used in the original analysis, we performed the search against a more recent snapshot of the database dated 2024/10. The differentially expressed proteins (FDR < 0.05) between the database versions had a high overlap (Figure 3D). However, there were also two differentially expressed proteins that were not quantified at all in the original study or in the reanalysis using the old version of the database but were statistically significant when using the more recent version of the database (Figure 3E). These included glutathione synthetase (GSS), which catalyzes the production of glutathione, and Ras-related protein Rab-1A (RAB1A), both of which showed higher abundance in the breast cancer subtypes compared to DCIS. Interestingly, increased glutathione level has been associated with aggressive breast cancer and higher rates of metastases,^40^ whereas RAB1A has been found to promote cell proliferation and migration.^41^

### Performance Metrics Allow Workflow Optimization

Nextflow provides several built-in performance metrics that can be used to monitor and analyze the execution of a workflow, including wall-clock time, CPU and memory usage, disk usage, and information on each task and their duration. These metrics can be explored and visualized with a web-based monitoring system.

We studied particularly the wall-clock time, CPU time, peak RAM, CPU usage, data set size (particularly the FASTA database), and the total number of spectra in the DIA files and in either the DDA or the pseudospectral files generated using glaDIAtor-nf (Table 1). Our analysis revealed that the number of DDA spectra or deconvoluted DIA spectra in the data set was the primary factor influencing the compute time (Pearson correlation coefficient *r* = 0.99, *p* < 0.01), with some influence also on the average CPU usage.

## Discussion

Systematic analysis of large and complex biological data, such as genomic or proteomic data, often requires a series of intricate steps that entail the use of various tools and techniques. Managing the complexity of these workflows is a well-established challenge and there has been a growing recognition of the need for effective workflow management in the field.^42,43^ We introduced here a free and open source implementation of the glaDIAtor workflow, designed for the untargeted analysis of DIA proteomics data on top of Nextflow workflow management system.^22^ This implementation is compatible with various high-performance computing platforms and can be readily installed and operated by users without prior programming experience.

We demonstrated high accuracy of glaDIAtor-nf on gold-standard benchmark data sets, with its sensitivity and specificity comparable to those of DIA-NN (Figure S1). Moreover, while performing multigroup differential expression analysis between different breast cancer subtypes in a clinical data set using ROTS identified 90 more differentially expressed proteins when it was called on the protein quantitation matrix of glaDIAtor-nf instead of the quantification matrix taken from the original publication. Notably, proteins with changes reported as significant only with the glaDIAtor-nf quantitation matrix included several well-known cancer-related proteins such as PARP1, THBS2, CAPZA2, and PRAF2, all of them showing elevated expression in the invasive breast cancer subtypes when compared to noninvasive DCIS. With these findings, our study underscores the significance of up-to-date software implementations and reanalysis of existing data sets to unveil previously undetected information.^44^

We found Nextflow to be an effective workflow management system with good integration capabilities for existing tools. Specifically, its compatibility with the various scheduling systems, such as SLURM,^45^ and container technologies, like Apptainer^46^ and Podman,^47^ makes a powerful combination in managing the software execution and execution environment, for example, libraries. We also found the Nextflow’s capability of monitoring resource usage in each step of the analysis useful, allowing for a detailed understanding of the resource requirements of the workflow and facilitating optimization of their bottlenecks. This is particularly important in proteomics data analysis workflows which tend to have long running times and high resource consumption. Finally, Nextflow is a domain specific language (DSL), built on top of Groovy programming language, providing an intuitive syntax for defining workflow steps in a clear and readable manner.

The current implementation of glaDIAtor-nf comes with a few limitations. The pipeline is currently based on the Nextflow DSL1 version which requires users to start the pipeline by explicitly invoking an older Nextflow engine version (22.10.1). Refactoring to DSL2 is considered as a priority for an upcoming release. Another notable limitation of the tool is that currently, timsTOF PASEF data is not supported.

The most computationally intensive aspect of the analysis of mass spectrometry proteomics data is typically the processing of the mass spectra, due to the massive amounts of data produced by mass spectrometers. Fortunately, however, the processing of this type of data is inherently parallelizable and can be executed at two levels. First, a single spectrum file contains multiple spectra that can be processed independently in parallel, referred to as thread-level parallelization, utilizing multiple processing cores of a single computer. Second, proteomics data sets typically consist of multiple spectrum files that can also be processed independently in parallel, whereby multiple computers can be utilized. The original implementation of the glaDIAtor workflow^22^ was able to utilize thread-level parallelization but only processed spectrum files in sequence, leading to extended analysis runtimes. In contrast, the Nextflow implementation glaDIAtor-nf is also capable of leveraging parallelization in computer clusters through scheduling systems.

In the untargeted scenarios involving only DIA data, estimating resource usage prior to deconvolution poses a challenge due to the uncertainty surrounding the number of spectra that a single spectrum might deconvolute into. This ambiguity complicates the accurate prediction of resource requirements before the deconvolution process.

The reproducibility of scientific experiments is a crucial attribute that must be maintained. In data processing, the selection of methods and software used can significantly affect the results, and this principle applies not only to proteomics but also to other scientific domains. Therefore, it is imperative to utilize a consistent and stable software environment to ensure that the results are reproducible. This can be achieved through the use of containers, which are self-contained computing environments that include everything necessary for a program to run. While container images are immutable, it can be difficult to recreate an identical image after a certain period of time, due to the constant updates in software repositories from traditional package managers. In this work, we utilized GNU Guix,^48^ which we have currently used to package the majority of software dependencies of glaDIAtor-nf in a reproducible manner, with possible future avenues covering the entire software stack that glaDIAtor-nf uses. GNU Guix guarantees the exact replication of the entire software environment in the container image when (re)­building a container, whether now or in the future. This can be done by the Guix *time-machine* command, which allows one to “time-travel” to how the world’s software sources were at a certain time.

Finally, the reliability of computational analysis is closely tied to the quality of the data. Having high-quality data allows accurate identification and quantification of proteins and generally increases reproducibility of results. In the context of DIA workflows, additional factors such as the spectral library influence data quality, and consequently, the success of downstream analyses. These aspects have been systematically assessed e.g., in a recent investigation of quality metrics for modern data-independent acquisition data^49^ and specifically on plasma and serum samples in a large multicenter study.^50^

To summarize, glaDIAtor-nf provides a modern workflow for the analysis of DIA mass spectrometry proteomics data, which is compatible with a wide range of computational platforms. Its open-source nature and foundation on a widely used workflow manager make it easily accessible to the community and open to contributions for further developments. Utilizing a workflow built on top of Nextflow allows for an easy replacement of components with newer alternatives emerging as the technologies develop, thereby promoting the longevity and adaptability of the workflow. Importantly, the vast publicly available archives of mass spectrometry data^51^ offer interesting prospects for reanalysis using new workflows, such as glaDIAtor-nf, with high potential for new insights^44^ and exploration of new research questions that were not covered in the original research.

## Supplementary Material



## Data Availability

Data are available via ProteomeXchange through the identifiers PASS00589 for the Bruderer data set PXD026600 for the Gotti data set, MSV000090837 for the Jumel data set and PXD014194 for the Valo data set. Code availability: The software is available from https://github.com/elolab/glaDIAtor-nf In addition, definitions of the software packaged for the GNU Guix package manager for the purposes of this workflow can be found as Guix channels under: https://github.com/elolab/proteomics-guix, https://github.com/elolab/proteomics-guix-nonfree.

## Notes

Performance comparison between glaDIAtor-nf and DIA-NN on gold-standard datasets (Figure S1); Principal component analysis and sample correlations of clinical proteomics data re-analyzed with glaDIAtor-nf (Figure S2); and DIA-NN reference sequences and run parameters (PDF)
