# Supplementary material for: Complete Data Analysis Workflow for Quantitative DIA Mass Spectrometry Using Nextflow
Source: J Proteome Res. 2026 Feb 6;25(3):1265–73. doi: 10.1021/acs.jproteome.5c00266 (PMC12973368; doi:10.1021/acs.jproteome.5c00266)
Supplement: Supplementary file 1 [file pr5c00266_si_001.pdf]

Complete data analysis workflow for quantitative DIA mass spectrometry using Nextflow

Mats Perk [1], Sami Pietilä [1], Tommi Välikangas [1],
Balazs Balint [1], Tomi Suomi [1,*,#], Laura L. Elo [1,2,*,#]

[1] Turku Bioscience Centre, University of Turku and Åbo Akademi University, FI-20520 Turku, Finland
[2] Institute of Biomedicine, University of Turku, FI-20520 Turku, Finland
* Correspondence: tomi.suomi@utu.fi, laura.elo@utu.fi
[#] Equal contribution

Table of contents

Figure S1: Performance comparison between glaDIAtor-nf and DIA-NN on gold-standard datasets

Figure S2: Principal component analysis and sample correlations of clinical proteomics data re-analyzed with glaDIAtor-nf

Supplementary Materials and Methods:
DIA-NN reference sequences and run parameters

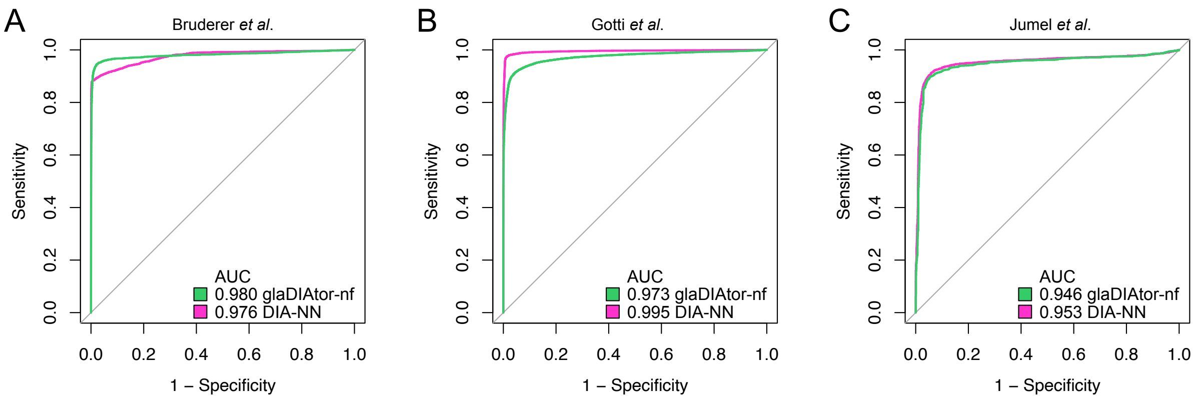

Figure S1: Performance comparison between glaDIAtor-nf and DIA-NN on three gold-standard datasets. Receiver operating characteristic (ROC) curves summarize the performance of glaDIAtor-nf (green) and DIA-NN (magenta) across all possible pairwise comparisons in the (**A**) Bruderer, (**B**) Gotti, and (**C**) Jumel datasets, together with the corresponding areas under the ROC curves (AUCs).

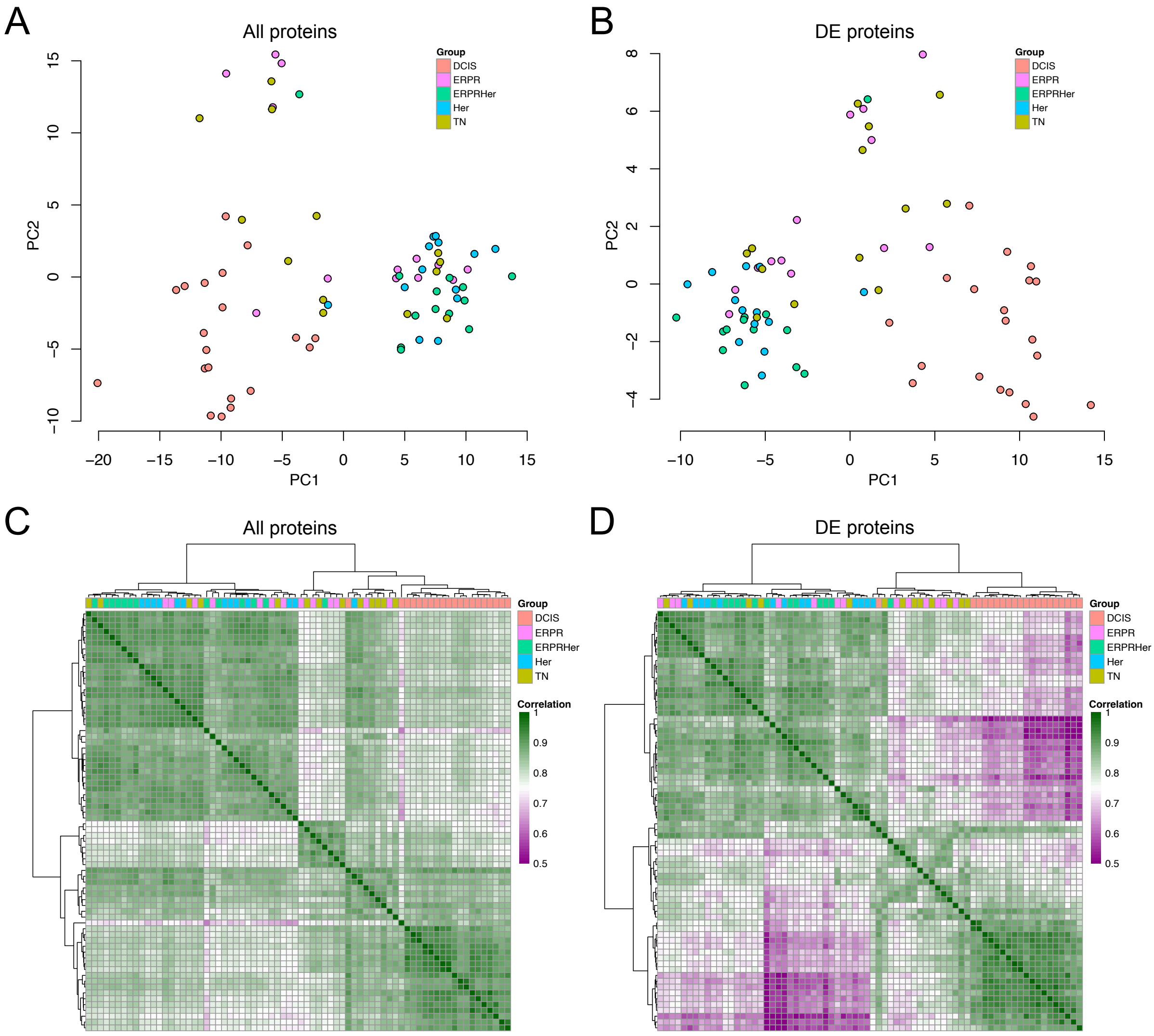

Figure S2: Principal component and correlation analysis of clinical proteomics data from Valo *et al*. re-analyzed using glaDIAtor-nf. PCA plots (**A**) on the full protein quantification matrix as well as (**A**) on the differentially expressed proteins (FDR < 0.05). Correlation analysis (**C**) on the full protein quantification matrix as well as (**D**) on the differentially expressed proteins (FDR < 0.05).

Supplementary Materials and Methods

DIA-NN reference sequences and run parameters

The UniProtKB database of Human (2017/04, 20183 protein entries supplemented with non-human proteins were used for the in *silico library* prediction as a reference for Bruderer data, UniProtKB database of *E.Coli* (2016/03, 4314 protein entries), combined with 48 UPS1 spike-in proteins was taken for Gotti data while the UniProtKB database of *E.Coli*, human, and yeast from the original publication (2020/08, 30810 total proteins) was used as reference for the Jumel data. DIA-NN was called with parameters "`--verbose 4 --fasta-search --min-fr-mz 200 --max-fr-mz 1800 --met-excision --cut K*,R* --missed-cleavages 1 --min-pep-len 7 --max-pep-len 30 --min-pr-mz 300 --max-pr-mz 1800 --min-pr-charge 1 --max-pr-charge 4 --qvalue 0.01 --matrices --max-pr-charge 4 --unimod4 --lib "" --gen-spec-lib --predictor --reanalyse --smart-profiling`".
